# Non-IgE-Mediated Hypersensitivity to Amoxicillin Following Epstein-Barr Virus Infection: A Case Report

**DOI:** 10.7759/cureus.93244

**Published:** 2025-09-26

**Authors:** Lara Margarida Navarro, Ana Isabel Moreira Ribeiro, Cláudia Patraquim, Joana Cunha de Oliveira

**Affiliations:** 1 Department of Pediatrics, Unidade Local de Saúde de Braga, Braga, PRT

**Keywords:** amoxicillin hypersensitivity, beta-lactam allergy, drug provocation test, epstein–barr virus infection, pediatric case report

## Abstract

In children with Epstein-Barr virus (EBV) infection, rash following amoxicillin use may resemble a viral exanthem, yet drug hypersensitivity must also be considered. We report the case of a previously healthy 13-month-old boy who developed a generalized erythematous maculopapular rash while on amoxicillin for otitis media and tonsillitis. EBV infection was confirmed by polymerase chain reaction. The rash resolved with supportive care. Three months later, the patient underwent allergy evaluation due to the severity of the initial eruption. Skin prick, intradermal tests, and specific IgE to beta-lactams were negative. However, four hours after an oral amoxicillin challenge, he developed periorificial and acral edema, consistent with a non-immediate hypersensitivity reaction. The challenge with cefuroxime was negative. This case highlights the potential of EBV to contribute as a cofactor in beta-lactam sensitization, even when the initial clinical presentation mimics a classical viral exanthem. Clinicians should consider reevaluation in children presenting with severe or atypical rashes during EBV infection, as drug provocation testing remains essential for distinguishing benign viral rashes from true allergic reactions.

## Introduction

Epstein-Barr virus (EBV) is a ubiquitous herpesvirus that is the primary cause of infectious mononucleosis, particularly in children and young adults. In this setting, administration of aminopenicillins is frequently associated with rash, which has traditionally been regarded as a benign, transient, non-allergic phenomenon, most often attributed to a virus-drug interaction rather than true hypersensitivity [1,2]. The reported incidence of rash in this context varies widely, with early reports suggesting rates of up to 90% among patients receiving amoxicillin during acute infectious mononucleosis [3]. Although many cases resolve without long-term implications, recent studies have highlighted that a subset of patients develop confirmed, persistent drug allergies, such as IgE-dependent or T-cell-mediated reactions [4-7].

The mechanisms underlying this phenomenon are not yet fully understood. Proposed hypotheses include EBV-induced immune dysregulation, polyclonal B- and T-cell activation, and altered drug metabolism, resulting in the enhanced presentation of drug antigens to the immune system [2,5]. In specific individuals, this immune activation may progress to a T-cell-mediated hypersensitivity reaction, which can be reproducibly triggered by re-exposure to the same antibiotic months or years later [6,7].

Distinguishing between transient viral exanthems and true drug hypersensitivity is clinically relevant, as mislabeling a patient with a beta-lactam allergy can lead to the use of broader-spectrum, less effective, and more costly antibiotics [1,3,6]. Accurate diagnosis relies on a careful clinical history, supported by allergologic workup, most notably supervised drug provocation testing, which remains the gold standard [1,5].

We report the case of a pediatric patient who developed a non-IgE-mediated amoxicillin hypersensitivity reaction in the setting of EBV infection, confirmed by positive drug provocation testing. This case highlights clinical scenarios in which post-infection reevaluation is critical to ensure both patient safety and antimicrobial stewardship.

## Case presentation

A 13-month-old previously healthy boy was admitted with fever, halitosis, and odynophagia. He had completed a 10-day course of amoxicillin for acute otitis media and tonsillitis two days before admission. On the last day of treatment, he developed a generalized maculopapular eruption, initially involving the face and trunk and progressing in a cephalocaudal distribution. The rash became increasingly confluent over the following days and was associated with significant pruritus. Two days later, he developed a high-grade fever (maximum 39.9°C), accompanied by reduced vitality, occasional cough, and a maternal report of halitosis.

**Figure 1:**
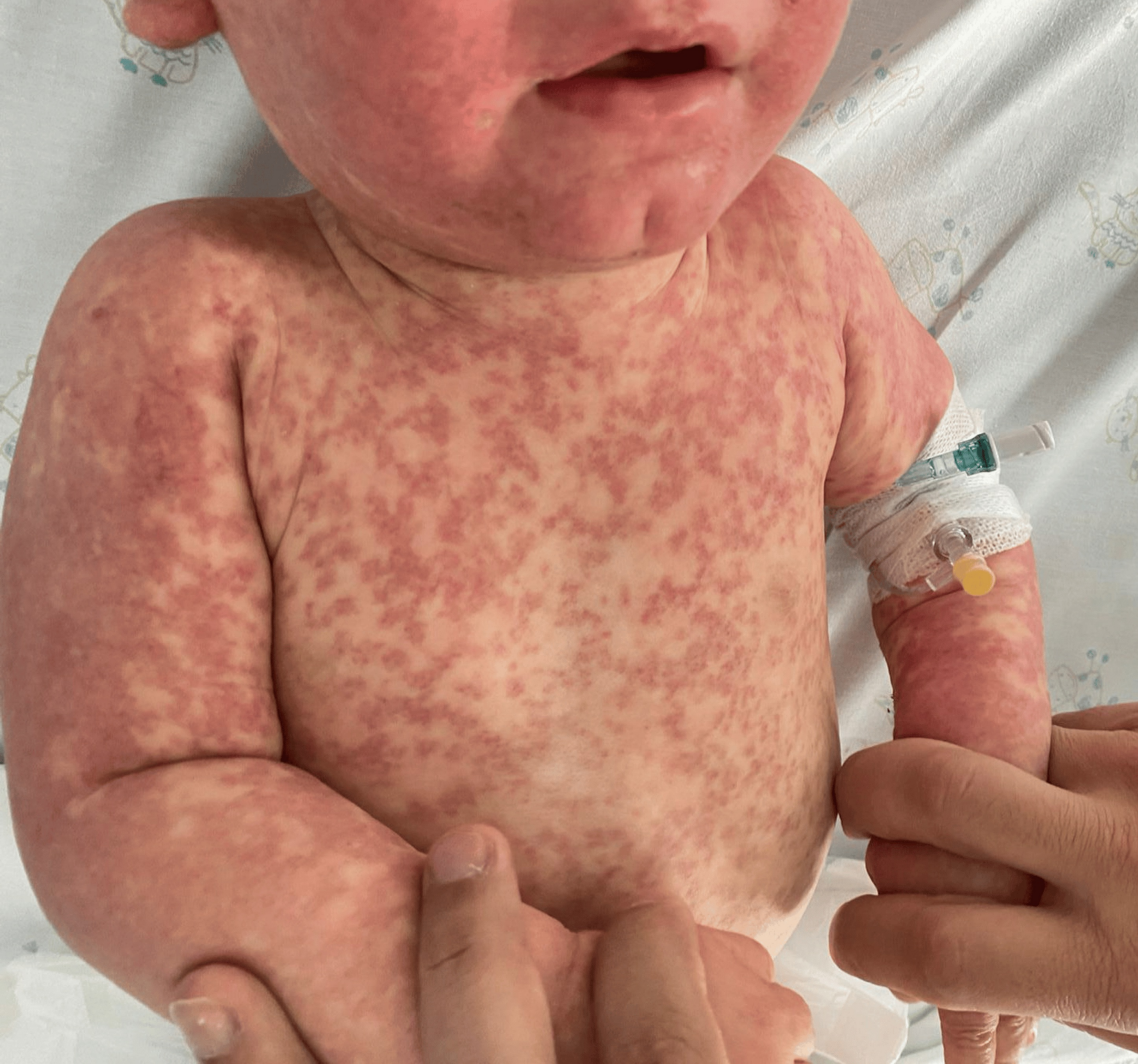
Dense, confluent erythematous maculopapular rash on the anterior trunk and upper limbs, with mucosal sparing, documented at hospital admission two days after completing a 10-day course of amoxicillin and shortly after rash onset The guardian consented to the publication of the patient's images in an open-access publication. A signed consent statement was provided to the journal.

**Figure 2:**
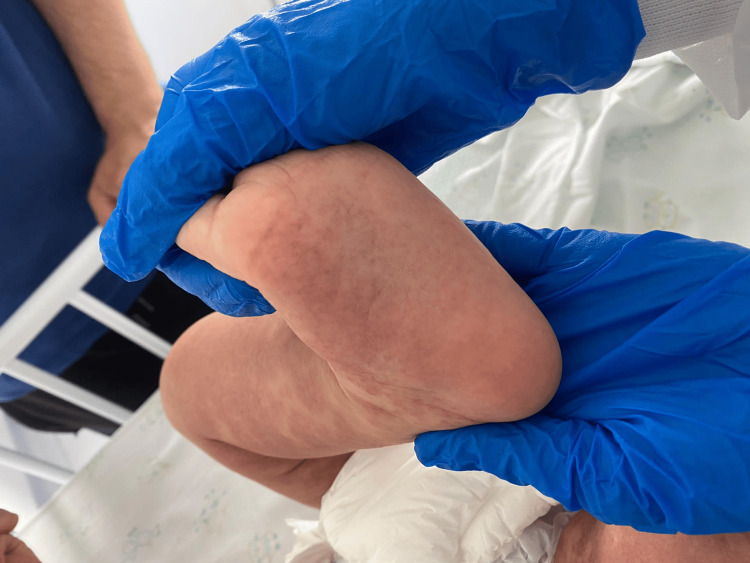
Erythematous macular and violaceous rash on the plantar region and posterior leg, not sparing pressure areas, observed at hospital admission two days after completing a 10-day course of amoxicillin, during the early phase of the eruption The guardian consented to the publication of the patient's images in an open-access publication. A signed consent statement was provided to the journal.

**Figure 3:**
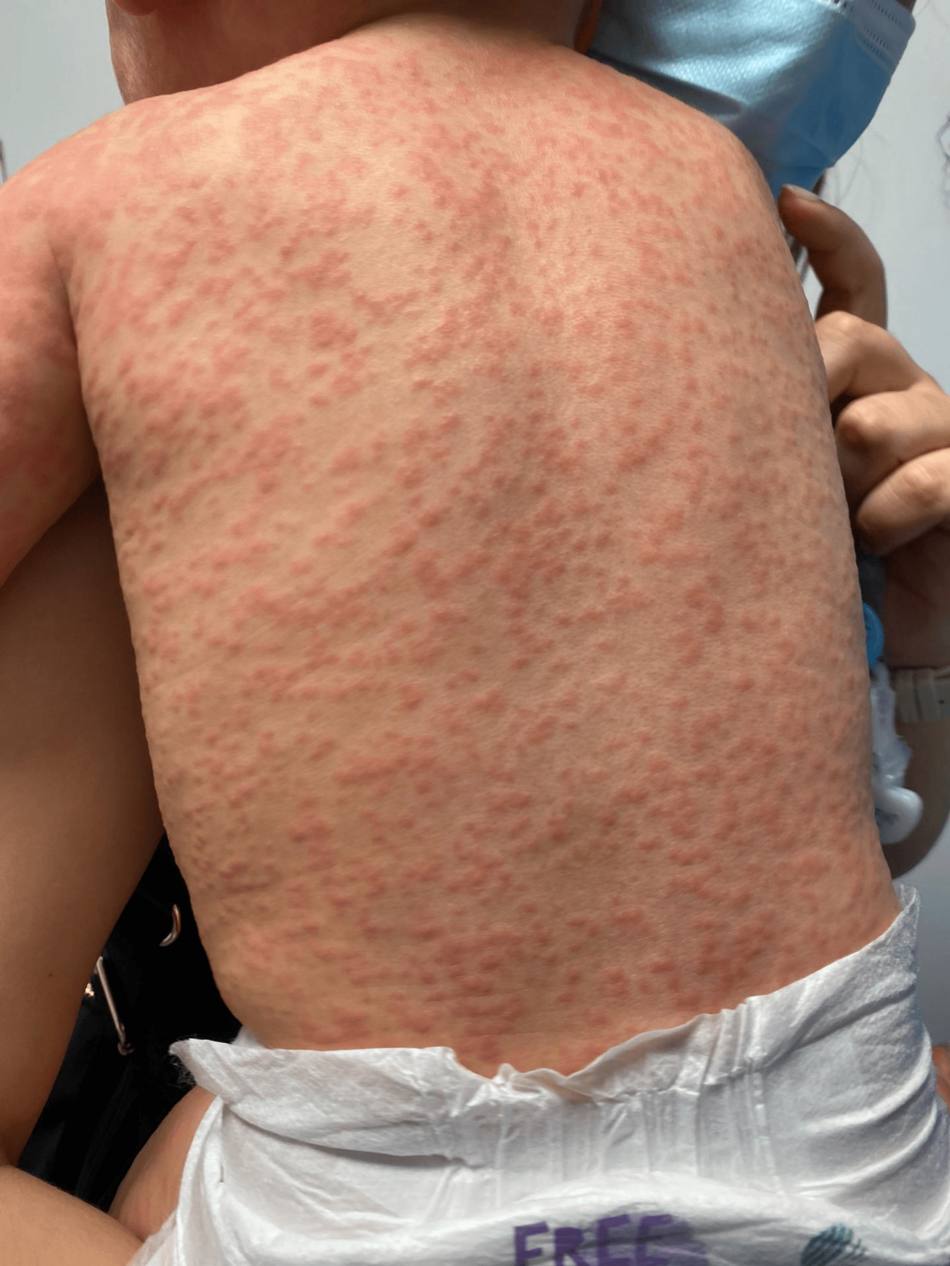
Confluent erythematous eruption on the dorsal trunk, recorded at hospital admission two days after completing a 10-day course of amoxicillin The guardian consented to the publication of the patient's images in an open-access publication. A signed consent statement was provided to the journal.

On examination, there was bilateral tonsillar hypertrophy and a diffuse, pruritic, erythematous maculopapular rash involving the face, trunk, and extremities, with no mucosal involvement (Figures 1-3). The eruption was confluent in some areas, particularly over the limbs and trunk. Non-tender inguinal lymphadenopathy was also noted, while hepatosplenomegaly was absent.

Laboratory evaluation revealed a normal total leukocyte count, but with 30% activated/reactive lymphocytes. C-reactive protein was mildly elevated, and liver and renal function tests were within normal limits. EBV infection was investigated at the time of admission (two days after the end of amoxicillin therapy). Serology results showed negative EBV early antigen IgG, negative viral capsid antigen IgG, equivocal viral capsid antigen IgM, and positive EBV nuclear antigen IgG. These findings were considered inconclusive, given the timing of the illness. A qualitative EBV PCR was subsequently performed six days after admission and was positive, confirming acute EBV infection. A complete list of laboratory and microbiological investigations performed during the acute phase is summarized in Table 1.

**Table 1:** List of complementary diagnostic tests during the acute phase AST: aspartate aminotransferase, ALT: alanine transaminase, EBV: Epstein-Barr virus, CMV: cytomegalovirus, DNA: deoxyribonucleic acid, IgG: immunoglobulin G, IgM: immunoglobulin M

Test	Result	Reference range
Complete blood count		
Hemoglobin	11.1	10.5-13.5 g/dL
Hematocrit	32.4	33-39%
Leukocytes	11300	600-17500/uL
Neutrophils	3800	1000-8500/uL
Lymphocytes	3400	4000-10500/uL
Reactive/activated lymphocytes	30%	-
Platelets	242000	>150000/uL
Erythrocyte sedimentation rate	14	1-15 mm/h
Blood chemistry		
Glucose	92	74-106 mg/dL
Urea	25	19-49 mg/dL
Creatinine	0.3	0.24-0.41 mg/dL
Sodium	136	136-145 mmol/L
Potassium	4.6	3.5-5.1 mmol/L
Chloride	105	98-107 mmol/L
AST	44	<50 U/L
ALT	23	7-40 U/L
C-reactive protein	7.50	<5 mg/L
Microbiology		
Hemoculture	Negative	-
DNA quantification (polymerase chain reaction)		
Enterovirus	Negative (stool)	-
EBV	Positive (blood)	-
Serologies		
IgG CMV	Positive (15.4 index)	Negative: <0.9
		Equivocal: 0.9-1.1
		Positive: >1.1
IgM CMV	Negative (0.346 index)	Negative: <0.9
		Equivocal: 0.9-1.1
		Positive: >1.1
IgG EBV early antigen	Negative (<<5.00)	Negative: <5 U/mL
		Equivocal: 5-10 U/mL
		Positive: >10 U/mL
IgG EBV viral capsid antigen	Negative (<10)	Negative: <20 U/mL
		Equivocal: 20-40 U/mL
		Positive: >40 U/mL
IgM EBV viral capsid antigen	Equivocal (25.4)	Negative: <20 U/mL
		Equivocal: 20-40 U/mL
		Positive: >40 U/mL
IgG EBV nuclear antigen	Positive (32.7)	Negative: <5 U/mL
		Equivocal: 5-20 U/mL
		Positive: >20 U/mL
Monospot	Negative	-
*Streptococcus* A antigen	Negative	-
IgG parvovirus B19	Negative (<<0.10 index)	Negative: <0.9
		Doubtful: 1-1.1
		Positive: >1.1
IgM parvovirus B19	Negative (<<0.10 index)	Negative: <0.9
		Doubtful: 1-1.1
		Positive: >1.1
IgG herpes I	Negative (0.12)	Negative: <0.9
		Doubtful: 1-1.1
		Positive: >1.1
IgM herpes I	Negative (<0.5)	Negative: <0.9
		Doubtful: 1-1.1
		Positive: >1.1
IgG herpes II	Negative (0.55)	Negative: <0.9
		Doubtful: 1-1.1
		Positive: >1.1
IgM herpes I	Negative (<0.5)	Negative: <0.9
		Doubtful: 1-1.1
		Positive: >1.1
IgM *Mycoplasma pneumoniae*	Negative	-

**Figure 4:**
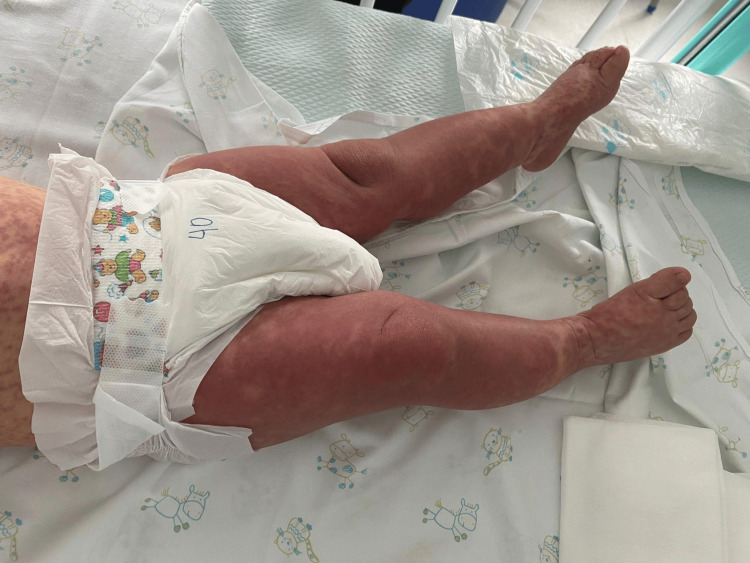
A marked confluent erythemato-violaceous rash on the lower limbs, involving the feet, was observed at hospital discharge after eight days of admission, corresponding to the late phase of the eruption The guardian consented to the publication of the patient's images in an open-access publication. A signed consent statement was provided to the journal.

During hospitalization, the rash regressed slowly but progressively. At discharge, after eight days of inpatient care, the patient still exhibited a macular erythematous-violaceous eruption (Figure 4), which was confluent on the limbs and trunk but lighter in intensity, involving the palms, soles, and face. He was discharged in good general condition with follow-up scheduled. At medical reevaluation one week after discharge, the exanthem had completely resolved.

Three months later, given the severity of the initial eruption, a comprehensive allergologic assessment was undertaken. Skin prick and intradermal tests for penicillin G, penicillin V, amoxicillin, and ampicillin were negative. Serum-specific IgE assays for these antibiotics were also negative (Table 2). An oral amoxicillin provocation test was then performed under hospital supervision, using three escalating oral doses up to a cumulative dose of 50 mg/kg administered at 60-minute intervals, followed by a two-hour observation period after the final dose. Four hours after the last administration, the patient developed hand edema, perianal erythema, and penile swelling. Symptoms resolved with oral corticosteroids and antihistamines, consistent with a non-IgE-mediated hypersensitivity to amoxicillin. A subsequent cefuroxime challenge was uneventful. The patient was advised to avoid aminopenicillins and was provided with a written plan outlining their allergy.

**Table 2:** Serum-specific IgE (RAST) results IgE: immunoglobulin E, RAST: radioallergosorbent test

Allergen	Result (kU/L)	Class	Reference range
Penicillin G	<0.10	0	Class 0: <0.35 kU/L, Class 1: 0.35-0.7 kU/L, Class 2: 0.7-3.5 kU/L, Class 3: 3.5-17.5 kU/L, Class 4: 17.5-50 kU/L, Class 5: 50-100 kU/L, Class 6: >100 kU/L
Penicillin V	<0.10	0	
Amoxicillin	<0.10	0	
Ampicillin	<0.10	0	

## Discussion

Although amoxicillin rashes in the setting of EBV infection are often non-allergic, a subset represents true hypersensitivity. Proposed mechanisms include EBV-induced polyclonal lymphocyte activation and promotion of drug-specific T-cell responses [1,2]. Reports describe persistent allergy confirmed by positive provocation or skin testing months after resolution of the viral illness [3-7]. In the present case, EBV serology at admission was inconclusive, which may be attributed to the early timing of testing, as antibody responses can be delayed in acute infections. In such situations, PCR provides greater diagnostic sensitivity and is ultimately confirmatory.

The temporal association, negative immediate hypersensitivity tests, and delayed reaction during oral provocation strongly suggest a T-cell-mediated mechanism. However, additional immune assays (e.g., lymphocyte transformation test) were not performed. This aligns with data showing that standard skin tests and specific IgE lack sensitivity for delayed reactions [1,5]. While transient rashes during EBV are common, the severity and distribution in this patient, coupled with the later reproducible reaction, support true sensitization rather than transient immunostimulation [6].

Clinically, this distinction matters: children with exuberant or atypical rashes during EBV treated with aminopenicillins should be reevaluated. Drug provocation testing under supervision remains the gold standard for confirming or excluding beta-lactam allergy [2,7]. In our case, cefuroxime tolerance suggested selective aminopenicillin hypersensitivity, which is important for antibiotic stewardship and the avoidance of unnecessarily broad-spectrum alternatives.

Our findings support the possibility that EBV may contribute as a cofactor in drug eruptions. In this context, viral infection has the potential to facilitate sensitization, leading to persistent hypersensitivity. This case highlights the importance of individualized assessment and careful re-challenge in select patients to inform safe future prescribing.

## Conclusions

This case illustrates that not all aminopenicillin-associated rashes during EBV infection are benign or self-limiting. In selected pediatric patients with severe or atypical eruptions, post-infection allergologic reassessment, including supervised drug provocation testing, is essential to distinguish transient viral exanthems from true drug hypersensitivity. Accurate diagnosis enables safe future prescribing, prevents unnecessary avoidance of beta-lactams, and promotes optimal antimicrobial stewardship. However, these conclusions are drawn from a single case report and should not be generalized without further supporting evidence.
